# Approach of Chronic Pelvic Pain with Top Flat Magnetic Stimulation

**DOI:** 10.1155/2023/9983301

**Published:** 2023-09-16

**Authors:** Benedetta Salsi, Giulia Ganassi, Graziella Lopopolo, Silvia Callarelli, Alessandra Comito, Irene Fusco, Pablo González Isaza

**Affiliations:** ^1^Division of Dermatology, Poliambulatorio San Michele, Reggio Emilia 42121, Italy; ^2^Division of Gynecology, Poliambulatorio San Michele, Reggio Emilia 42121, Italy; ^3^Studio Medicò Surgical Laser, Pescia 51017, Italy; ^4^El.En. Group, Calenzano 50041, Italy; ^5^Hospital Universitario San Jorge Private Practice, CLINIEM Madrid Spain Pereira, Pereira, Colombia

## Abstract

**Materials and Methods:**

Vulvar Functional Status Questionnaire (VQ) was used for the evaluation of patient's chronic pelvic pain and muscle hypertone improvements. The interstitial cystitis was assessed by the Leary–Sant symptom and problem indexes (ICSI and ICPI). In this study, the scores resulting from the sum of the two indexes were evaluated as OSS (ICSI + ICPI).

**Results:**

Women with chronic pelvic pain and muscle hypertone showed VQ mean values significantly lower than the controls (*p*  <  0.005) from the second treatment up to the sixth one. In 6 patients affected by interstitial cystitis, the mean score of OSS was significantly lower than the controls (*p*  <  0.005) from the second treatment up to 2 months follow-up after the last treatment session. No side effects were observed.

**Conclusion:**

Based on these results, this technology may successfully manage muscle hypertonicity condition, the chronic pelvic pain, and interstitial cystitis.

## 1. Introduction

Pelvic floor tension myalgia (PFTM) is a type of pelvic floor dysfunction related to increased activity or hypertonicity of pelvic floor muscles that leads to chronic pelvic pain or miofascial syndrome [1]. It is uncomfortable and potentially harmful to have tight, constantly contracted pelvic floor muscles. Women experience this condition much more frequently than male patients in their fourth to sixth decade of life [2, 3]. The study of Mathias et al. [4] of more than 5,000 women aged 18 to 50 found that 14.7% of them had chronic pelvic pain that lasted for more than six months. It is important to keep in mind that pelvic pain syndromes are the root cause of sexual dysfunction. Early referral for sexual counseling during treatment is frequently beneficial for couples [5].

The exact cause of pelvic floor tension myalgia is not known, but multiple variables may contribute to its development, such as nerve damage, irritable bowel syndrome, endometriosis, genitourinary inflammation, interstitial cystitis, poor posture, aging, obesity, history of sexual abuse, pudendal nerve entrapment, trauma, reaction to pelvic organ disease, postsurgical scarring, rectal disorders, central pain sensitization, and psychological factors. Pelvic floor muscle spasm is thought to be a major cause of PFTM. Recurrent urinary tract infections (rUTIs), in particular interstitial cystitis symptoms without infection, are common in women and frequently present in general practice [6, 7].

Interstitial cystitis, also known as painful bladder syndrome, can cause lower abdominal pain/discomfort, dyspareunia, and pain in any lower spinal nerve distribution. Additional bladder-related symptoms, such as frequency, urgency, or nocturia, are experienced by patients with this condition. It can affect people of all ages and should be considered for people who have other chronic pain conditions such as fibromyalgia, irritable bowel syndrome, chronic fatigue, or chronic pelvic pain [8].

Interstitial cystitis/painful bladder syndrome is frequently associated with high-tone pelvic floor dysfunction. Because of their syndromic nature and amorphous definitions, these conditions are common in gynaecological patients presenting with chronic pelvic pain and are frequently misdiagnosed. Clinicians should be cautious of these processes in patients who suffer from recurrent urinary tract infection symptoms or chronic pelvic pain [9]. The two common tests used to diagnose interstitial cystitis are urinalysis/urine culture and cystoscopy, as reported by the National Institute of Diabetes and Digestive and Kidney Diseases.

Generally, a gynaecologist with great expertise follows a therapeutic path aimed at reducing the three triggering causes of pelvic chronic pain condition: reduction of local inflammation as not to further fuel the disease, regularization of nerve transmission to decrease pain, and relaxation of contracted muscles (to improve the condition of the vulvar tissue, reduce friction during sexual intercourse and abrasions on the mucous membranes, and the consequent postcoital infections and irritation).

Treatment for PFTM is frequently difficult, with numerous treatments showing limited success. The literature on PFTM treatment is quite varied. Massage, medication, high-voltage electro-galvanic stimulation (HVGS), biofeedback, short-wave diathermy, botulin toxin injections, relaxation therapies, cognitive behavioral therapy, ultrasound, sit baths, posture training, hydrotherapy, strengthening exercises, and transcutaneous electrical nerve stimulation (TENS) units appear to be most beneficial in these patients. The outcomes of these treatments varied, and there is not much evidence to support or refute their efficacy [10]. Analgesics and muscle relaxants are often employed to treat pelvic floor tension myalgia. Analgesics may reduce pain, but they do not address the underlying abnormality. Diazepam has been widely used, but only temporary improvement is obtained. Unfortunately, there are women who do not respond to conservative therapies represented by medical/pharmacological management, physical therapy techniques (electromyography (EMG) biofeedback, manual therapy, or electrotherapy), and surgery [11, 12]. In addition, patients are naturally reluctant to consider invasive surgical treatment [13]. Even localized TENS [14, 15] (therapy applied generally for genital syndrome and vulvar pain) does not appear to be effective for chronic pelvic pain treatment, as the vaginal musculature is too contracted to fit a probe and pain can be exacerbated.

Magnetic stimulation has been explored as an alternative to electrical stimulation recently for urological diseases, clinical neuro-diagnostic applications, and pelvic floor muscle strengthening, pain, and hypertonia to provide safe and noninvasive treatment for chronic pelvic pain syndrome [16–18].

Recently, Biondo and Colleagues [19] observed that patients reported significant improvement of vulvar pain as a result of PISQ-12's domain changes following top flat magnetic stimulation technology.

Based on these considerations, our study provided insight into the assessment of the effectiveness and safety of a novel device that uses top flat magnetic stimulation to treat women with interstitial cystitis and chronic pelvic pain.

## 2. Materials and Methods

### 2.1. Patient Population

A total of 12 Caucasian women with a mean age of 30.66 (±14.66) were enrolled for this study (see Table 1).

All (12/12) patients presented with chronic pelvic pain such as dyspareunia, pain during intercourse, and vestibulodynia, 4/12 patients presented muscle hypertone and 6/12 patients presented with interstitial cystitis (with negative urinoculture).

To perform a correct and accurate diagnosis of chronic pelvic pain, we have excluded all the pathologies which could be responsible for the same type of painful symptoms; exclusion criteria included the presence of patients with menopause state, pelvic organ prolapses, genital infections, menstruation, malignant tumors, severe neurological diseases, pregnancy, obesity, and persons with pacemakers or metal implants. Inclusion criteria included patients affected by chronic pelvic pain and pelvic floor hypertonicity.

The diagnosis of hypertonic pelvic floor is performed in all patients by an experienced gynaecologist with a manual assessment of the pelvic floor muscles and a swab test.

This study was conducted in accordance with the Good Practice Guideline and the Declaration of Helsinki and informed consent was obtained from all patients.

### 2.2. Study Device

A noninvasive electromagnetic therapeutic device (DR ARNOLD, DEKA M.E.L.A. Calenzano, Italy) with a main unit and a chair applicator was used during the procedures. The coil of the chair applicator, which is located in the center of the seat, is intended for therapy of the deep pelvic floor area. The patient is seated on the chair with their perineum on the center of the seat, which helps them feel the stimulation of their pelvic floor and sphincter muscles during stimulation therapy. In order to stimulate the pelvic area, DR. ARNOLD can produce an electromagnetic field with a homogenous profile (TOP FMS-TOP Flat Magnetic Stimulation). Gynaecologists initially set and adjusted the patient's position before each session to ensure an appropriate level of stimulation and achieve a uniform distribution of muscle involvement. The beneficial effect of the subject device is due to a greater uniformity of magnetic field distribution over a larger area, which allows for greater recruitment of muscle fibers without generating areas that differ in stimulation intensity. The device's change in magnetic fields transmits the current into nerves which send signals to the muscles in order to contract and release them. The crucial thing is the positioning of the patient (patient's pelvic floor muscle (PFM)) in the chair. Patients PFM should be positioned in the center of the magnetic field for optimal (maximal) effect. First and foremost, the patient's comfort throughout the lengthy treatment session is ensured. Second, it makes it possible to center the electromagnetic field on the patient's perineum. With the study device, the patient can be positioned precisely so that his legs are in the right positions by adjusting the seat height prior the treatment. The legs of the patient are positioned perpendicularly, ensuring the thighs are parallel to the floor with the feet flat on the ground. At the knee, patients ought to create an angle of 90 degrees or slightly higher. The subject system is equipped with protocols for reducing hypertonicity that use low-frequency stimulations (around 10 Hz) in accordance with the treatment's goals.

### 2.3. Study Protocols

The DR ARNOLD system was applied for a total of 6 treatments on all patients. Sessions were conducted twice weekly for three consecutive weeks; depending on the patient's muscular condition, each session lasted 28 minutes. The overtone/pain protocol (muscle work aimed at muscle inhibition and reduction of pain (hyperactivity and hypertone)) was selected after the first two minutes of warm-up for all patients. Data were collected at baseline, after each treatment session up to 6 treatments, and after 2 months of follow-up after the last treatment session. In all patients, chronic pelvic pain was evaluated using the Vulvar Functional Status Questionnaire (VQ) [20].

The interstitial cystitis was assessed by the Leary-Sant symptom and problem indexes (ICSI and ICPI). In this study, the scores resulting from the sum of the two indexes were evaluated as OSS (ICSI + ICPI) [21, 22].

Possible side effects and adverse events such as muscular pain, temporary muscle spasm, temporary joint/tendon pain, or local erythema/skin redness were evaluated during all treatment periods.

SPSS (IBM Corp., New York, USA) was used for all statistical analyses. Descriptive statistical analysis (means and ±SDs) was performed using the Student's *t*-test. A *p*  <  0.05 is considered to be significant.

## 3. Results

**Figure 1:**
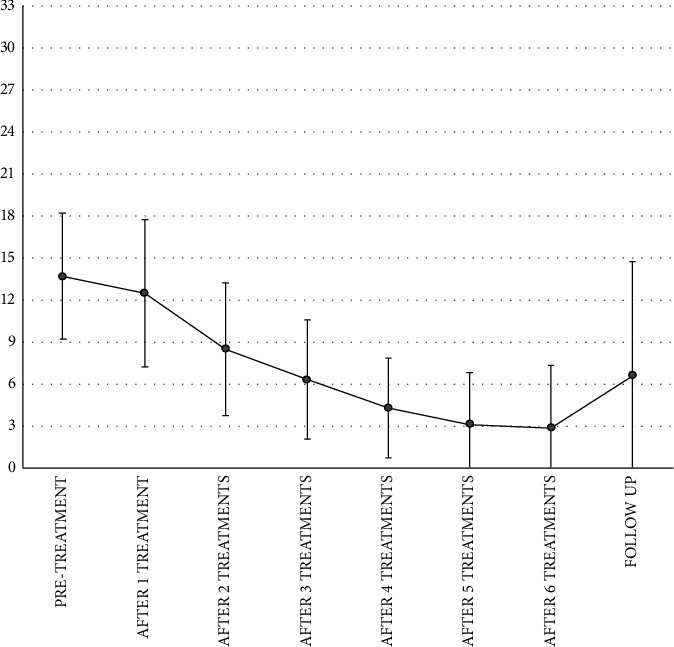
Vulvar Functional Status Questionnaire (VQ) mean scores at baseline, after each treatment session up to 6 treatments, and after 2 months of follow-up after the last treatment session.

Women with chronic pelvic pain alone or in association with muscle hypertonicity showed VQ mean values significantly lower than the controls (*p*  < 0.005) from the second treatment up to the last one. For all patients evaluated, the mean score of Vulvar Functional Status Questionnaire (VQ) decreased from 13.7 (±4.47) at baseline to 12.5 (±5.25) after 1 treatment session, significantly decreased from 13.7 (±4.47) at baseline to 8.5 (±4.74) after 2 treatment sessions, to 6.3 (±4.24) after 3 treatment sessions, to 4.3 (±3.56) after 4 treatment sessions, and to 3.1 (±3.67) after 5 treatment sessions and to 2.86 (±4.49) after 6 treatment sessions, showing an improvement in chronic pelvic pain and in the four patients with hypertonicity symptoms (Figure 1). The VQ mean score increases at 2 months after the last treatment compared to the score revealed at the sixth treatment but remains below the baseline value. The nonsignificance of the reduction of the VQ score at two months of follow-up compared to the baseline can be explained by the fact that a small number of patients were considered (5/12 patients**).**

**Figure 2:**
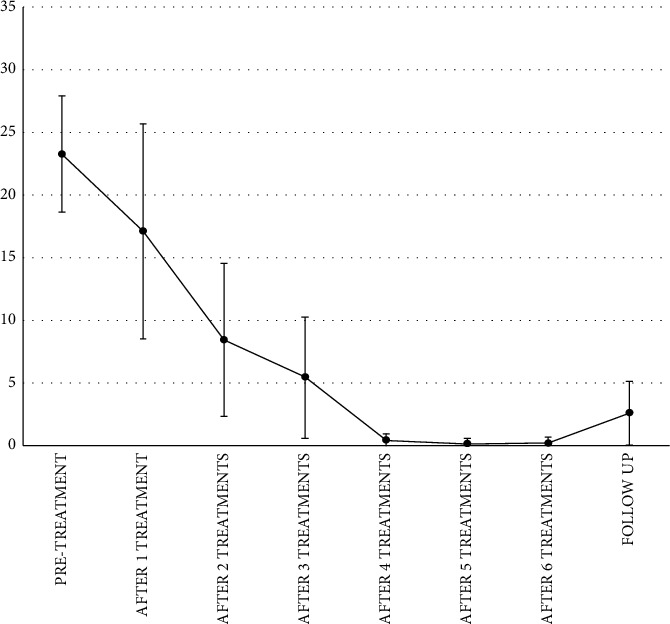
Representation of OSS mean scores at baseline, after each treatment session up to 6 treatments, and after 2 months of follow-up after the last treatment session.

In 6 patients affected by interstitial cystitis, the mean score of OSS decreases from 23.3 (±4,63) at baseline to 17.2 (±8,59) after 1 treatment session, significantly decreases from 23.3 (±4,63) at baseline to 8.5 (±6.16) after 2 treatment sessions, to 5.5 (±4.85) after 3 treatment sessions, to 0.5 (±0.55) after 4 treatment sessions, to 0.2 (±0.45) after 5 treatment sessions, to 0.25 (±0.5) after 6 treatment sessions, and to 2.6 (±2.52) at 2 months follow-up after the last treatment session (Figure 2). No side effects were observed.

## 4. Discussion

The pelvic floor muscles are deeply stimulated by magnetic stimulation technology, which restores neuromuscular control [23]. Muscle contraction or relaxation, neuronal cell depolarization, and effects on the blood circulation system are all outcomes of the interaction with the tissue. The scientific literature [24–36] states that this technology can affect sexuality and health in a broad patient population and safely and effectively treat stress, urge, and mixed urinary incontinence by strengthening the pelvic floor muscles. The severity of UI symptoms was less severe, and the subjects used absorbent pads less frequently, both of which improved their quality of life. Patients also reported additional therapeutic effects, such as improved urination control and increased sexual satisfaction, based on the subjective evaluation. Nevertheless, since the subject device can also select a specific protocol for hypertonicity, the great advantage is the ability to treat pathologies such as chronic pelvic pain and muscle hypertonicity thanks to its decontracting action. Indeed, the overtone/pain protocol for hypertonic management may employ lower frequencies (around 10 Hz) to generate a homogenous electromagnetic field distribution that does not create regions of different stimulation intensity, avoiding overstimulation of the already hypersensitive receptors and sensory nerves typical of chronic pelvic pain. Our findings showed an improvement in the patient's chronic pelvic pain (such as dyspareunia, pain during intercourse, and vestibulitis) and muscle hypertone symptoms as assessed by VQ, without finding any side effects. The VQ is a valid questionnaire with sufficient internal consistency, test-retest reliability, and validity to be useful for women's health physical therapists [20].

Women with chronic pelvic pain alone or in association with muscle hypertonicity showed VQ mean values significantly lower than the controls (*p*  <  0.005) from the second treatment up to the last one.

We have observed that the VQ mean score increases slightly two months after the last treatment when compared to the score revealed at the sixth treatment, but it remains lower than the baseline value. The nonsignificance of the reduction of the VQ score at two months of follow-up compared to the baseline can be explained by the fact that a small number of patients were considered (5/12 patients). However, there are already published studies [32] in which an increase in the scores of the questionnaires used for the evaluation of urinary incontinence is observed with longer follow-ups.

In addition, interstitial cystitis was also treated with success as assessed by OSS questionnaire which reported promising results. In addition, when compared to the assortment of devices available for pelvic floor restoration, the subject device offers additional significant benefits. It is a noninvasive device first and foremost because no probe is inserted into the vaginal channel during muscle stimulation. As a result, patients can remain fully clothed in a supportive and ergonomic chair and resume their daily activities right away after sessions thanks to the consistent emission of energy that is gradually delivered. Finally, the DR. ARNOLD system can be viewed as an “educator” system, since it allows the patient to feel the relaxation of the muscles that are being treated, giving them more autonomy and awareness to decide when to repeat the next treatment session.

In addition, this technology can be utilized in conjunction with other pharmacological or physical techniques [30]. Our long-term goal is to enroll more patients applying longer follow-up, in order to further investigate this innovative and noninvasive method of treatment for complicated pathologies such as muscle hypertonicity, chronic pelvic pain, and interstitial cystitis.

### 4.1. Study Limitation

Study limitations are represented by a small patients' sample, a short follow-up, and the lack of a control group.

## 5. Conclusions

Our investigation reveals that this technology could represent a promising approach for the treatment of chronic pelvic pain as well as muscle hypertonicity condition and interstitial cystitis. Based on 2-month follow-up data, it seems that the effect is short-lasting, but a limited number of patients were evaluated at this time. In order to monitor the device's long-term effects, additional studies with follow-ups of up to 4 or 6 months will be required.

## Figures and Tables

**Table 1:** Patients' demographic information.

Number of patients	Race	Gender	Age (years)
1	Caucasian	Female	19
2	Caucasian	Female	44
3	Caucasian	Female	24
4	Caucasian	Female	22
5	Caucasian	Female	17
6	Caucasian	Female	29
7	Caucasian	Female	23
8	Caucasian	Female	68
9	Caucasian	Female	28
10	Caucasian	Female	25
11	Caucasian	Female	24
12	Caucasian	Female	45

## Data Availability

The data used to support the findings of this study are available from the corresponding author upon request.

## References

[1] Faubion S. S., Shuster L. T., Bharucha A. E. (2012). Recognition and management of nonrelaxing pelvic floor dysfunction. *Mayo Clinic Proceedings*.

[2] Thiele G. H. (1963). Coccygodynia: cause and treatment. *Diseases of the Colon & Rectum*.

[3] Sinaki M., Merritt J. L., Stillwell G. K. (1977). Tension myalgia of the pelvic floor. *Mayo Clinic Proceedings*.

[4] Mathias S. D., Kuppermann M., Liberman R. F., Lipschutz R. C., Steege J. F. (1996). Chronic pelvic pain: prevalence, health-related quality of life, and economic correlates. *Obstetrics and Gynecology*.

[5] Goldstein A. T., Pukall C. F., Brown C. (2016). Vulvodynia: assessment and treatment. *The Journal of Sexual Medicine*.

[6] McKertich K., Hanegbi U. (2021). Recurrent UTIs and cystitis symptoms in women. *Australian Journal of General Practice*.

[7] Hanno P. M., Erickson D., Moldwin R., Faraday M. M. (2015). Diagnosis and treatment of interstitial cystitis/bladder pain syndrome: AUA guideline amendment. *The Journal of Urology*.

[8] McLennan M. T. (2014). Interstitial cystitis: epidemiology, pathophysiology, and clinical presentation. *Obstetrics and Gynecology Clinics of North America*.

[9] Moody C. C., Fashokun T. B. (2021). Painful bladder syndrome/interstitial cystitis and high tone pelvic floor dysfunction. *Obstetrics and Gynecology Clinics of North America*.

[10] Meister M. R., Sutcliffe S., Badu A., Ghetti C., Lowder J. L. (2019). Pelvic floor myofascial pain severity and pelvic floor disorder symptom bother: is there a correlation?. *American Journal of Obstetrics and Gynecology*.

[11] Dionisi B., Anglana F., Inghirami P., Lippa P., Senatori R. (2008). Use of transcutaneous electrical stimulation and biofeedback for the treatment of vulvodynia (vulvar vestibular syndrome): result of 3 years of experience. *Minerva Ginecologica*.

[12] Ghisu G. P. (1994). Vulvodynia diagnostics and management strategies. *Praxis*.

[13] Edwards L. (2015). Vulvodynia. *Clinical Obstetrics and Gynecology*.

[14] Waldinger M. D., de Lint G. J., Venema P. L., van Gils A. P. G., Schweitzer D. H. (2010). Original research—women’s sexual health: successful transcutaneous electrical nerve stimulation in two women with restless genital syndrome: the role of a*δ*- and C-nerve fibers. *The Journal of Sexual Medicine*.

[15] Murina F., Felice R., Di Francesco S., Oneda S. (2018). Vaginal diazepam plus transcutaneous electrical nerve stimulation to treat vestibulodynia: a randomized controlled trial. *European Journal of Obstetrics and Gynecology and Reproductive Biology*.

[16] Galloway N. T., El-Galley R. E., Sand P. K., Appell R. A., Russell H. W., Carlan S. J. (1999). Extracorporeal magnetic innervation therapy for stress urinary incontinence. *Urology*.

[17] Yamanishi T., Sakakibara R., Uchiyama T. (2000). Comparative study of the effects of magnetic versus electrical stimulation on inhibition of detrusor overactivity. *Urology*.

[18] Rowe E., Smith C., Laverick L., Elkabir J., Witherow R. O. ’N., Patel A. (2005). A prospective, randomized, placebo controlled, double-blind study of pelvic electromagnetic therapy for the treatment of chronic pelvic pain syndrome with 1 year of followup. *The Journal of Urology*.

[19] Biondo A., Murina F., Fusco I. (2022). Treatment of pelvic floor hypertonic disorders with top flat magnetic stimulation in women with vestibulodynia: a pilot study. *Journal of Women’s Health and Development*.

[20] Hummel-Berry K., Wallace K. (2007). Reliability and validity of the vulvar functional Status questionnaire (VQ). *Journal of Women’s Health Physical Therapy*.

[21] O’Leary M. P., Sant G. R., Fowler F. J., Whitmore K. E., Spolarich-Kroll J. (1997). The interstitial cystitis symptom index and problem index. *Urology*.

[22] Kuo Y.-C., Kuo H.-C. (2015). O’Leary-Sant symptom index predicts the treatment outcome for OnabotulinumtoxinA Injections for refractory interstitial cystitis/bladder pain syndrome. *Toxins*.

[23] Gilling P. J., Wilson L. C., Westenberg A. M. (2009). A double-blind randomized controlled trial of electromagnetic stimulation of the pelvic floor vs sham therapy in the treatment of women with stress urinary incontinence. *BJU International*.

[24] Unsal A., Saglam R., Cimentepe E. (2003). Extracorporeal magnetic stimulation for the treatment of stress and urge incontinence in women--results of 1-year follow-up. *Scandinavian Journal of Urology and Nephrology*.

[25] Yokoyama T., Inoue M., Fujita O., Nozaki K., Nose H., Kumon H. (2005). Preliminary results of the effect of extracorporeal magnetic stimulation on urinary incontinence after radical prostatectomy: a pilot study. *Urologia Internationalis*.

[26] Choe J. H., Choo M.-S., Lee K.-S. (2007). Symptom change in women with overactive bladder after extracorporeal magnetic stimulation: a prospective trial. *International Urogynecology Journal*.

[27] Vadalà M., Palmieri B., Malagoli A., Laurino C. (2018). High-power magnetotherapy: a New weapon in urinary incontinence?. *Lower Urinary Tract Symptoms*.

[28] Chandi D. D., Groenendijk P. M., Venema P. L. (2004). Functional extracorporeal magnetic stimulation as a treatment for female urinary incontinence: ’the chair. *BJU International*.

[29] Cidranes D., Blanco E. (2018). Safety and preliminary efficacy of magnetic stimulation of pelvic floor with hifem technology in urinary incontinence. *Medical and Clinical Research*.

[30] Rosen N. O., Dawson S. J., Brooks M., Kellogg-Spadt S. (2019). Treatment of vulvodynia: pharmacological and non-pharmacological approaches. *Drugs*.

[31] González-Isaza P., Sánchez-Borrego R., Lugo Salcedo F. (2022). Pulsed magnetic stimulation for stress urinary incontinence and its impact on sexuality and health. *Medicina*.

[32] Lopopolo G., Salsi B., Banfi A., Isaza P. G., Fusco I. (2022). Is it possible to improve urinary incontinence and quality of life in female patients? A clinical evaluation of the efficacy of top flat magnetic stimulation technology. *Bioengineering*.

[33] Isaza P. G., Borrego R. S., Fusco I. (2022). A case of stress urinary incontinence after radical prostatectomy successfully treated with an innovative device based on top flat magnetic stimulation. *World Journal of Urology*.

[34] Dominguez A. P., Isaza P. G., Pantoja S. N., Fusco I. (2022). Role of top flat magnetic stimulation for urinary incontinence as a debilitating condition of pelvic floor dysfunction: an observational evaluation of Latin American population. *World Journal of Urology*.

[35] Biondo A., Gonzalez Isaza P., Fusco I. (2022). Efficacy of top flat magnetic stimulation technology for female stress and urge urinary incontinence: a clinical evaluation. *World J Nephrol Urol*.

[36] Frigerio M., Barba M., Cola A. (2023). Flat magnetic stimulation for stress urinary incontinence: a prospective comparison study. *Bioengineering*.

